# Comparative innocuity and efficacy of live and inactivated sheeppox vaccines

**DOI:** 10.1186/s12917-016-0754-0

**Published:** 2016-06-29

**Authors:** Zineb Boumart, Samira Daouam, Imane Belkourati, Lamya Rafi, Eeva Tuppurainen, Khalid Omari Tadlaoui, Mehdi El Harrak

**Affiliations:** Research and Development Virology, Multi-Chemical Industry, Lot. 157, Z I, Sud-Ouest (ERAC) B.P.: 278, Mohammedia, 28810 Morocco; Capripoxvirus Reference Laboratory, The Pirbright Institute, Ash Road, Pirbright, Woking, Surrey GU24 0NF United Kingdom

**Keywords:** Sheeppox, Romanian strain, Inactivated vaccine, Efficacy, Potency

## Abstract

**Background:**

Sheeppox (SPP) is one of the priorities, high-impact animal diseases in many developing countries, where live attenuated vaccines are routinely used against sheeppox virus (SPPV). In an event of an SPP outbreak, historically disease-free countries would hesitate to use of live vaccines against SPPVdue to the safety and trade reasons. Currently no killed SPPV vaccines are commercially available. In this study, we developed an inactivated Romanian SPPVvaccine and assessed its efficacy and potency in comparison with a live attenuated Romanian SPPV vaccine. Four naïve sheep were vaccinated once with the Romanian SPPV live attenuated vaccine and16 sheep were vaccinated twice with the inactivated vaccine. All sheep in the live vaccine group were included in the challenge trial, which was conducted using a highly virulent Moroccan SPPV field strain. Eight sheep of the inactivated vaccine group were challenged and the remaining sheep were monitored for seroconversion. Experimental animals were closely monitored for the appearance of clinical signs, body temperature and inflammation at the injection site. Two naïve sheep were used as unvaccinated controls.

**Results:**

The inactivated Romanian SPPV vaccine was found to be safe and confer a good protection, similar to the live vaccine. Specific antibodies appeared from seven days post vaccination and remained up to nine months.

**Conclusion:**

This study showed that the developed inactivated Romanian SPPV vaccine has a potential to replace attenuated vaccine to control and prevent sheep pox in disease-free or endemic countries.

## Background

Sheeppox virus (SPPV), the etiological agent of sheeppox (SPP), is a member of the genus *Capripoxvirus* within the family *Poxviridae*. SPP is a transboundary disease which is notifiable to the World Organization of Animal Health (OIE) [1]. The disease can be mild in indigenous sheep breeds, but usually causes severe or fatal infection in newly introduced, fully susceptible animals. In naïve animals, morbidity and mortality may be as high as 100 % [2]. SPPV affects all ages of sheep but the clinical signs are more severe in young lambs [3]. The disease is characterized by fever, generalized papules or nodules in the skin, respiratory distress, pox lesions in the respiratory and gastrointestinal tracts, and sometimes death. SPP is endemic in Central and North Africa, the Middle East, Central Asia and the Indian subcontinent. Recently, SPP has also reached southern Europe [4].

In endemic countries, vaccination is considered the only economically feasible way to control the disease and improve small ruminant productivity. Numerous live vaccines have been developed and worldwide used, while inactivated vaccines are considered less effective and have only been tested at the laboratory level [5]. Despite the high efficacy of live vaccines, SPP is still endemic in those regions where vaccination is routinely practiced. In North Africa where SPPV has a strict sheep tropism, after decades of vaccination using a live attenuated vaccine, SPP is still causing significant economic losses for sheep farming industry [6]. In Greece, SPP re-occurred in 2013 and in 2015 the outbreak is still continuing, despite extensive stamping-out of infected and in-contact animals, movement restrictions and other supportive control and eradication measures (OIE Wahid database). The use of SPPV live vaccine is not permitted within the European Union (EU) member states. However, in a situation like in Greece, a safe non-replicating, effective and cheap inactivated emergency vaccine could provide a more efficient tool to limit the spread of the disease without a need for culling a large number of animals which is expensive and highly stressful for the farmers, destroying decades of work for the genetic improvement of a flock.

According to current OIE trade recommendations and EU directive (90/425/EEC of 26 June 1990), both SPP outbreak and use of any SPPV vaccines would inflict immediate restrictions to the export of live animals and their products from affected to disease-free countries. In addition, three years after the last SPP case or last vaccination, are required to re-gain the disease-free status. The lack of DIVA component in currently available vaccines would hamper the retrospective serological surveys, although it is unlikely that the antibody levels in vaccinated animals would remain on the detectable level for three years.

In another hand, it has been reported that some attenuated live vaccines induced severe pock reactions at the injection site or even mild disease in vaccinated animals [7]. Poor quality live vaccines (low vaccine titre) may also serve as vehicles for extraneous virus contaminants such as Border Disease virus (BDV). In the past, outbreak of BDV occurred inTunisia after vaccination by a BDVcontaminated SPPV vaccine and in France abovine viral diarrhea (BVD) contaminated Aujeszky disease live vaccine [8, 9]. In addition, theoretically there is a possibility that an attenuated vaccine virus could revert back to virulent, although such an occurrence has never been reported in SPPV vaccines.

Thus, an inactivated SPPV vaccine would provide a safe and valuable tool to protect livestock against SPPV, particularly during the first incursion of the virus in the previously disease-free country. In this study, we developed an inactivated vaccine against SPPV and tested its efficacy and potency by serology and challenge experiment in comparison with a live attenuated SPPV vaccine.

## Methods

### Vaccine preparation

The SPPV Romanian strain was propagated on Vero cells [10] and maintained in Dulbecco’s Modified Eagle’s Medium (DMEM) with 10 % irradiated fetal calf serum. The inoculation was carried out using an M.O.I (Multiplicity of Infection) of 0.01.

The live vaccine was prepared from the virus suspension by the addition of stabilizer (4 % peptone, 8 % sucrose and 2 % glutamate) followed by lyophilization. The inactivated vaccine was prepared from the same virus suspension by inactivating the virus with β-propiolactone. The complete inactivation of the vaccine virus and sterility of the product was confirmed before vaccine formulation. The aluminum hydroxide adjuvant was added at 2.1 mg/ml. The final product was distributed in 50 ml vials and stored at 4 °C before use.

### Sheep vaccination

Eighteen healthy sheep, six to eight months of age, representing the Timehdit breed of the Atlas mountains, were tested SPPV seronegative by virus neutralization (VN) test. Group 1 (G1) comprised 16 sheep which were vaccinated subcutaneously in the groin area with a volume of 2 ml of the inactivated SPPV vaccine representing a dose of 10^5.5^ TCID_50_. Two sheep were kept as unvaccinated controls. Group 2 (G2) comprised four animals which were vaccinated with 0.5 ml of the live attenuated vaccine, representing a dose of 10^3.0^ TCID_50_. All immunized animals were daily monitored for 14 days for rise in body temperature, appearance of clinical signs typical for SPP and inflammation at the injection site. Animals in G1, received a booster vaccination at D21 in the same conditions. Serum samples were collected from the vaccinated sheep weekly until 2 months, then at one month interval for the remaining unchallenged eight sheep up to 9 months.

### Experimental challenge infection

Vaccine potency testing was carried out by a challenge trial, using a virulent field SPPV strain (Hd2012) at the BSL3 laboratory. In the inactivated vaccine group G1, eight sheep out of 16 were challenged on D35 and in live vaccine group G2 four sheep were challenged on D28 after vaccination. Two naïve sheep were challenged as unvaccinated controls.

The challenge was conducted using a local Moroccan highly virulent SPPV field strain. The virus was administered by intra-dermal (ID) route in the flank of the animals at ten fold dilutions (10^-1^ to 10^-6^) to perform a virus titration comparatively on vaccinated and control animals.

Sheep were monitored daily for clinical signs, rectal temperature and the development of inflammation in each of the injection site. The presence of any inflammation was considered positive for the virus titration. The average virus titres of G1 and G2 were compared with the titre obtained in the unvaccinated animals and the difference between the two titres, expressed in log_10_, represented the protection index [11].

### Serological response

Serum samples were tested for the development of specific SPPV antibodies using VN test as described in the OIE Terrestrial Manual (OIE Chapters 2.7.11 and 2.7.14). This test is based on a serial ¼ dilutions of heat inactivated sera and a set amount of infectious virus (100 TCID_50_). The neutralizing antibody titer was calculated in accordance to Reed and Muench method [12].

### Statistical analyses

Differences between antibody titers obtained with live and inactivated vaccines, and between virus titers in vaccinated and unvaccinated animals, were determined using a one-way analysis of variance (ANOVA) followed by a student t-test. Values of *P* ≤0.05 were considered significant.

## Results

### Sheep vaccination

Three weeks following vaccination, the body temperature of vaccinated sheep in G1 remained within normal limits and no clinical signs typical for SPP were observed. At the vaccination site, transitory inflammation 1 cm to 2 cm in diameter was observed in some animals, disappearing in few days. In G2, slight increase in body temperature was observed six to eight days after vaccination and in some animals a local reaction was observed atthe vaccination site, persisting in some cases up to 20 days.

**Fig. 1:**
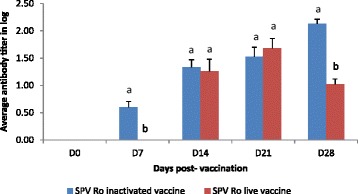
Neutralizing SPPV antibody titers after sheep vaccination. The results correspond to the mean antibody titers ± SEM of 16 sheep vaccinated with the inactivated Romanian SPPV vaccine strain (G1) and 4 sheep vaccinated with the live attenuated Romanian SPPV vaccine (G2). (a–b) indicate a significant difference in means antibody titers between animals of G1 and G2 at the 0.05 level

In G1, anti SPPV antibodies appeared as early as D7 post-vaccination. Compared to live vaccine, they registered similar values at D14 and D21, but reached a significantly higher value of antibody neutralizing titer (2.1 log_10_) on D28 (*P* ≤0.05). In G2, the rise of anti SPPV antibodies was noted later on D14 post-vaccination, and showed a slight decrease on D28 to reach a value of 1 log_10_ (Fig. 1).

**Fig. 2:**
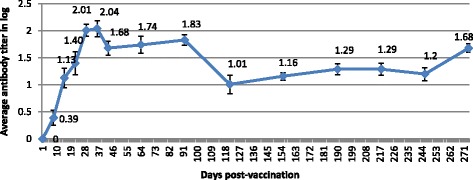
Neutralizing SPPV antibody titers after vaccination of sheep with the inactivated Romanian SPPV vaccine strain. The results correspond to the mean of antibody titer of eight vaccinated sheep ± SEM

Most vaccinated sheep in G1showed an increase of antibody titre following the booster. In eight unchallenged vaccinated sheep, the immunity persisted for at least 9 months. The antibody neutralizing titre seemed to stabilize at 1 to 2.1 log_10_ (Fig. 2).

### Experimental infection

**Fig. 3:**
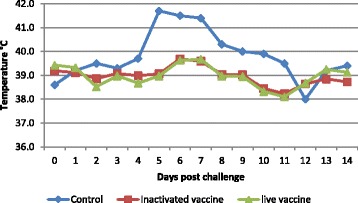
Temperature monitoring of unvaccinated and vaccinated (G1 and G2) sheep during 14 days post-challenge

**Fig. 4:**
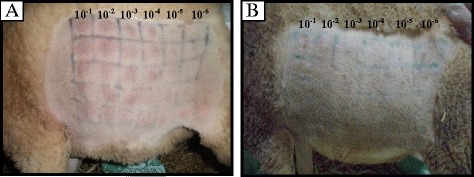
**a** Figure of challenged unvaccinated sheep showing local inflammations on site of inouclation (flank) with 10^–1^ to 10^–6^ dilutions (left to right) of virulent SPPV in five replica. **b** Challenged vaccinated sheep showing no local inflammations on the injection sites

On the challenge, the two unvaccinated control animals exhibited a rise in body temperature between D4 and D11 (Fig. 3). Local reactions at the injection sites were observed from D3, increasing in size the following days. Typical SPPV skin nodules (not associated with injection sites) appeared between D10 and D11 (Fig. 4). The obtained virus titres on the flank of the two unvaccinated control animals were 5.5 log_10_ ID50/ml and 5.9 log_10_ ID50/ml, significantly higher than that obtained with vaccinated animals (*P* ≤ 0.05) (Table 1).

**Table 1:** Challenge results in control and vaccinated sheep with live and inactivated Romanian SPPV vaccine. Antibody neutralizing titers of vaccinated sheep obtained before challenge. The infectious titer represents the maximum value obtained between day 6 and 8 post challenge

Vaccine	Animals	Hypersensitivity titer in TCID_50_		Antibody neutralizing titers in log_10_		Infectious titer (ID_50_/ml)		Protection value	
		Per animal	Group average	Per animal	Group average	Per animal	Group average	Per animal	Group average
Control animals	299	0	0	0	0	5.2	5,4^(a)^	0	0^(a)^
	277	0		0		5.6		0	
Live SPPV vaccine	977	1,6	1,6	1,9	1,7	1,5	0,75^(b)^	3,9	4,6^(b)^
	928	1,6		1,26		0,5		4,9	
	934	1,4		1,98		0,5		4,9	
	999	1,8		1,5		0,5		4,9	
Inactivated SPPV vaccine	948	2,7	2,2	1,98	2,2	2,1	1,4 ^(b)^	3,3	4 ^(b)^
	941	0		2,22		2,5		2,9	
	949	4,5		2,46		0,5		4,9	
	397	1,1		1,98		0,5		4,9	
	398	2,5		2,46		0,5		4,9	
	998	0,9		1,74		0,5		4,9	
	973	4,5		2,46		0,5		4,9	
	969	1,5		2,7		4,1		1,3	

(a–b) indicate a significant difference in means of infectious titers and protection values between vaccinated and unvaccinated animals and between G1 and G2 at the 0.05 level

After the challenge, the vaccinated sheep of G1 and G2 showed a transient two days increase in temperature between 39.6 °C and 39.7 °C at D6 and D7(Fig. 3) and a hypersensitivity reaction at the injection site two days after the challenge, generally in the first dilutions.

None of the immunized animals showed clinical signsof SPP during the observation period. Inflammations due to the virus replication were observed in dilutions 10^-1^ and 10^-2^ between D4 and D12. There was no significant difference in the titer and the protection index between G1 and G2 (P ≥ 0.05). The obtained infectious titre was 1.4 log10 ID_50_/ml for the inactivated vaccine G1 and 0.75 log_10_ ID_50_/ml for the live vaccine G2 (Table 1). The protection index was estimated to 4 for G1 and 4.6 for G2.

All vaccinated animals of G1 and G2 showed anantibody response that increased after the challenge.

## Discussion

In endemic countries a variety of attenuated live vaccines have been used against SPPV. Live attenuated vaccine protection is mediated by both cellular and humoral immunity. While, inactivated vaccines are believed to be less effective in stimulating the cell mediated immune response, which is the predominant protective response to poxvirus infection. In addition, in vaccinated sheep a poor correlation between antibody levels and immune status of animals has been reported [5], suggesting that live replicating vaccines are required for the development of effective immunity against pox disease [13, 14].

Nevertheless, in many countries, live vaccines use was banished because of its potential to induce mild diseases in animals and the risk of contamination by extraneous pathogens. The use of inactivated vaccines would be, thus, an alternative to protect livestock against pox diseases. In the present study, an inactivated and live attenuated SPPV vaccines, were compared in terms of their safety and potency. Romanian strain has been selected for vaccine preparation because it proved to be effective for protection against SPP infection [10, 15].

Sheep were observed for three weeks following immunization. The inactivated vaccine was safe to use, as all vaccinated animals remained healthy, no increase in body temperature after vaccination was detected and only a small inflammation at the injection site was observed. A serological response was detected one week earlier for the inactivated vaccine group than for the live attenuated vaccine. This is in agreement with the previous studies, showing an increase in antibody titres between D7 and D21 post-vaccination and a boost of the immune response following a challenge [16, 17]. Neverthless, the need of a booster for inactivated vaccines is usually a constraint compared to live attenuated vaccine that need only one shot. Thus, it will be interesting, in a further study, to assess the vaccine protection using a single vaccination with killed vaccine.

The kinetic of antibody response in vaccinated sheep showed that inactivated vaccine provide detectable antibody levels for more than nine months, suggesting that an annual booster with this vaccine is enough to maintain a good protection at the population level. The long term duration of the protection needs to be confirmed using a challenge experiment. The findings of the present study are in agreement with other studies, demonstrating the efficacy of the inactivated SPP vaccines to protect sheep against challenge [17, 18].

The challenge was conducted according to the protocol defined by Fassi-Fehri and co-workers [11]. This method allows quantitative assessment of the conferred immunity and it is based on the obtained titres of the challenge virus in vaccinated and control animals. The difference of 2 log titers is used to determine the protection threshold. The method provides a protection index that is high as the immune response is strong and durable. Despite the high titres of the virulent challenge virus, both inactivated and live SPPV vaccines protected experimental animals against generalization of the disease observed in unvaccinated sheep. A short increase in body temperature in both vaccinated groups was observed for two days, which can be a physiological response triggered by any antigen. In contrast, unvaccinated sheep exhibited characteristic clinical signs, primary and secondary pox lesions with high fever between D4 and D11 post-infection with the challenge virus. The obtained virus titre in unvaccinated control animals reached 10^5.7^ID50/mlwhich is the normal titre of the virulent strain in sheep.

Inflammation due to the virus replication was observed only in low dilutions for both groups with lower infectious titres if compared to control animals, giving evidence of the virulent virus neutralization by the conferred immunity. There was no significant difference between live and inactivated vaccines after the challenge as indicated by the protection index values (4.6 versus 4.0). These values are comparable to those normally obtained with other live vaccines [19] and are in compliance with the OIE procedure of testing the vaccine potency taking a difference of log_10_ titre > 2.5 as evidence of protection [20].

## Conclusion

There are only limited studies on inactivated SPP vaccines and very few reports comparing the efficacy of live and attenuated vaccines [17, 18]. The current study showed that the developed inactivated Romanian SPPV vaccine is as potent as live vaccine and has a potential to replace attenuated vaccine to control and prevent sheep pox in disease-free or endemic countries, especially in those countries where the use of live vaccine is unauthorized. Inactivated vaccine is completely safe in all animals, does not present any risk of diffusion, reversion or extraneous pathogens spread.

## Abbreviations

SPP, sheeppox; SPPV, sheeppox virus; DIVA, differentiating infected from vaccinated animals; TCID_50_, tissue culture infective dose; MOI, Multiplicity of Infection; VN, virus neutralization
